# Supplementary material for: Does Music Intervention Improve Anxiety in Dementia Patients? A Systematic Review and Meta-Analysis of Randomized Controlled Trials
Source: J Clin Med. 2023 Aug 24;12(17):5497. doi: 10.3390/jcm12175497 (PMC10488399; doi:10.3390/jcm12175497)
Supplement: Supplementary file 1 [file jcm-12-05497-s001.zip › S1.pdf]

List of excluded articles

No	First Author / Publication Year	Title	Journal/Book	Exclusion
1	Alonso 2019	Treatment and control of behavioral and psychological symptoms	Recent Advances in Alzheimer Research	Not RCT
2	Creese et al., 2019	The development and use of the assessment of dementia awareness and person-centred care training tool in long-term care	Dementia	Not RCT
3	Curran., Loi S. 2012	Depression and dementia	Medical Journal of Australia	Not RCT
4	Svansdottir et al., 2006	Music therapy in moderate and severe dementia of Alzheimer's type: a case-control study	International Psychogeriatrics	Not RCT
5	Valdiglesias et al., 2017	Is Salivary Chromogranin A a Valid Psychological Stress Biomarker During Sensory Stimulation in People with Advanced Dementia?	Journal of Alzheimer's Disease	Not RCT
6	Davison et al., 2016	A personalized multimedia device to treat agitated behavior and improve mood in people with dementia: A pilot study	Geriatric Nursing	Pilot study
7	Hutson et al., 2014	Sonas: a pilot study investigating the effectiveness of an intervention for people with moderate to severe dementia	Am Journal of Alzheimer's Disease Other Demen	Pilot study
8	Thornley et al., 2016	Music therapy in patients with dementia and behavioral disturbance on an inpatient psychiatry unit: results from a pilot randomized controlled study	International Psychogeriatrics	Pilot study
9	Lai et al., 2016	Interdisciplinary collaboration in the use of a music-with-movement intervention to promote the wellbeing of people with dementia and their families: Development of an evidence-based intervention protocol	Nursing & Health Sciences	Protocol
10	Loi et al., 2022	Music and Psychology & Social Connections Program: Protocol for a Novel Intervention for Dyads Affected by Younger-Onset Dementia	Brain Sciences	Protocol
11	Mahendran et al., 2017	Art therapy and music reminiscence activity in the prevention of cognitive decline: Study protocol for a randomized controlled trial	Trials	Protocol
12	Tan et al., 2018	Study protocol for a randomized controlled trial of choral singing intervention to prevent cognitive decline in at-risk older adults living in the community	Frontiers in Aging Neuroscience	Protocol
13	Tang et al., 2013	The effectiveness of nursing management on improving health outcomes for hospitalized older adults with delirium: A systematic review protocol	JBI Database of Systematic Reviews and Implementation Reports	Protocol
14	Gaviola et al., 2020	Impact of individualised music listening intervention on persons with dementia: A systematic review of randomised controlled trials	Australasian journal on ageing	Review
15	Na et al., 2019	A systematic review and meta-analysis of nonpharmacological interventions for moderate to severe dementia	Psychiatry Investigation	Review
16	Noone et al., 2019	Meta-analysis of psychosocial interventions for people with dementia and anxiety or depression	Aging & mental health	Review
17	Scott S. et al., 2016	A scoping review of music and anxiety, depression and agitation in older people with dementia in residential facilities and specialist care units	European Geriatric Medicine	Review
18	Bakerjian et al., 2020	The Impact of Music and Memory on Resident Level Outcomes in California Nursing Homes	Journal of the American Medical Directors Association	Unrelated
19	Buard et al., 2021	Randomized controlled trial of neurologic music therapy in Parkinson's disease: research rehabilitation protocols for mechanistic and clinical investigations	Trials	Unrelated
20	Dell'Olio et al., 2020	Preoperative receptive music therapy in oral oncologic surgery: Preliminary study	Dental Cadmos	Unrelated
21	Giaquinto et al., 2006	Effects of music-based therapy on distress following knee arthroplasty	British Journal of Nursing	Unrelated
22	Kwak et al., 2020	Findings From a Prospective Randomized Controlled Trial of an Individualized Music Listening Program for Persons With Dementia	Journal of Applied Gerontology	Unrelated
23	McCreedy et al., 2022	Pragmatic Trial of Personalized Music for Agitation and Antipsychotic Use in Nursing Home Residents With Dementia	Journal of the American Medical Directors Association	Unrelated
24	Pérez-Ros et al., 2019	Preoperative receptive music therapy in oral oncologic surgery: Preliminary study	Journal of Alzheimer's Disease	Unrelated
25	Raglio et al., 2015	Effect of Active Music Therapy and Individualized Listening to Music on Dementia: A Multicenter Randomized Controlled Trial	Journal of the American Geriatrics Society	Unrelated
26	Yeh et al., 2019	Online Simulation-Based Mastery Learning with Deliberate Practice: developing Interprofessional Communication Skill	Clinical simulation in nursing	Unrelated
27	Young et al., 2019	Effects of Physical Activity Intervention for Chinese People With Severe Mental Illness	Research on social work practice	Unrelated
