# Supplementary material for: Does Music Intervention Improve Anxiety in Dementia Patients? A Systematic Review and Meta-Analysis of Randomized Controlled Trials
Source: J Clin Med. 2023 Aug 24;12(17):5497. doi: 10.3390/jcm12175497 (PMC10488399; doi:10.3390/jcm12175497)
Supplement: Supplementary file 1 [file jcm-12-05497-s001.zip › S2.pdf]

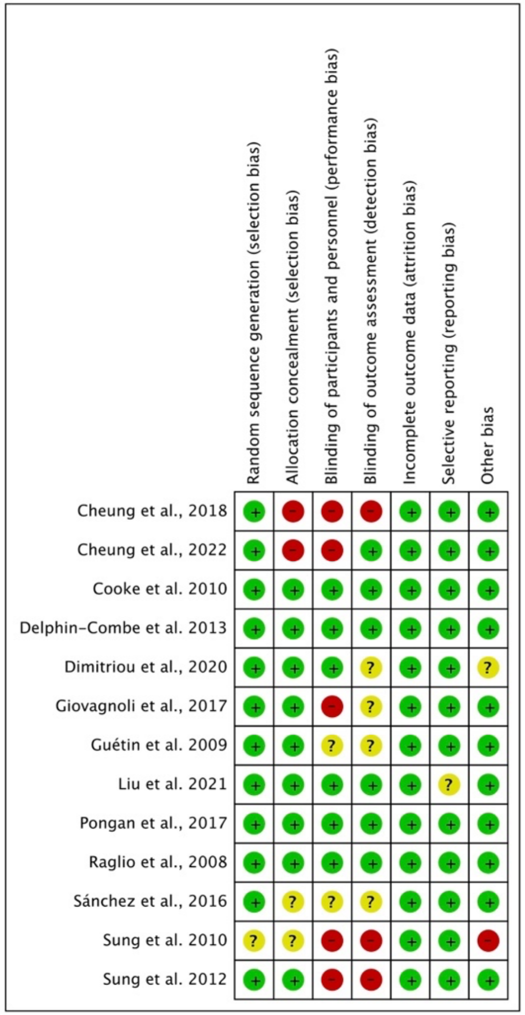

Figure S1a. Risk of bias summary: review authors' judgements about each risk of bias item for each included study.

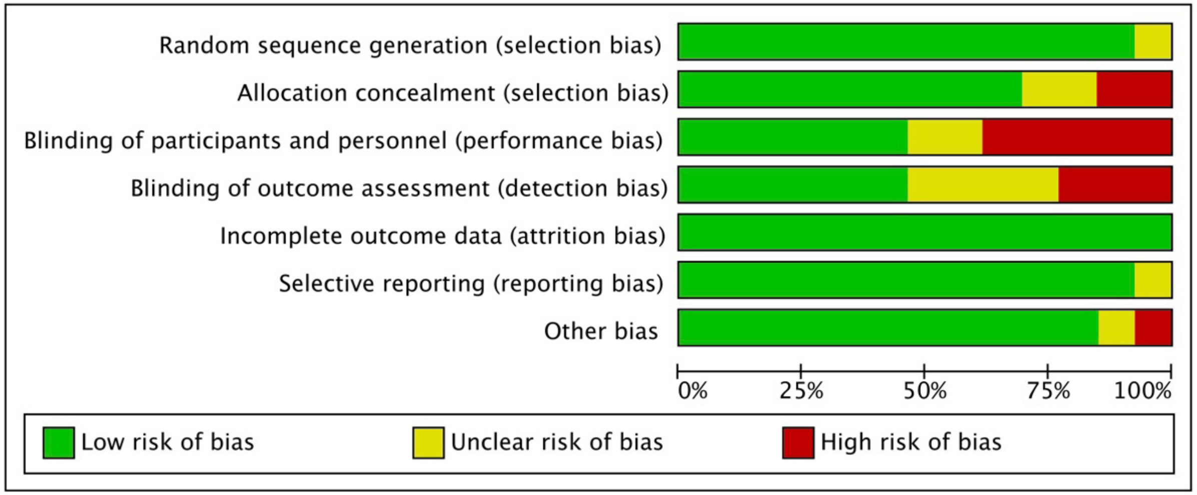

Figure S1b. Percentage of the risk of bias for each study.
